# When dermoscopy of a nodule on the hysterectomy scar reveals peritoneal carcinomatosis from high-grade serous tubal carcinoma

**DOI:** 10.1093/omcr/omaf303

**Published:** 2026-05-24

**Authors:** Kaoutar Benchekroun, Maryam Ghaleb, Salim Gallouj, Ouiame Eljouari

**Affiliations:** Dermatology Department, Mohammed VI University Hospital, Faculty of medicine and pharmacy, Abdelmalek Essaâdi University Tangier 90000, Morocco; Dermatology Department, Mohammed VI University Hospital, Faculty of medicine and pharmacy, Abdelmalek Essaâdi University Tangier 90000, Morocco; Dermatology Department, Mohammed VI University Hospital, Faculty of medicine and pharmacy, Abdelmalek Essaâdi University Tangier 90000, Morocco; Dermatology Department, Mohammed VI University Hospital, Faculty of medicine and pharmacy, Abdelmalek Essaâdi University Tangier 90000, Morocco

**Keywords:** cutaneous metastasis, hysterectomy scar, tubal carcinoma, peritoneal carcinomatosis, Dermoscopy, polymorphous vascular pattern

## Abstract

Cutaneous metastases are rare but clinically relevant manifestations of internal malignancies, occurring in fewer than 10% of cases and in only a small fraction of gynecologic cancers. They most commonly arise from ovarian carcinoma, while tubal carcinoma rarely produces such lesions. Their occurrence on a surgical scar is particularly unusual and may result from intraoperative tumor seeding or the permissive microenvironment of scar tissue, characterized by neoangiogenesis, impaired barrier function, and reduced local immunity. We present the case of a woman with high-grade serous tubal carcinoma whose recurrence began as a painful nodule and papules on the hysterectomy scar, later extending into infiltrated nodules across the lower abdomen and thigh roots. Dermoscopy revealed a diffuse erythematous background with polymorphic vascular structures, including serpentine vessels. Biopsy confirmed cutaneous metastasis. This case emphasizes dermoscopy’s diagnostic role in rare scar-associated cutaneous metastases.

## Introduction

Cutaneous metastases (CM) are uncommon but important in oncology, affecting fewer than 10% of patients with solid tumors [1]. They occur in approximately 2% of gynecologic malignancies, most often from ovarian carcinoma, and only rarely from tubal carcinoma [2, 3].

Metastases arising on surgical scars are exceptional. Proposed mechanisms include direct intraoperative tumor seeding and the permissive biological environment of scar tissue, characterized by increased vascularization and reduced immune resistance [4].

Clinically, CM are polymorphic and may mimic benign conditions, leading to diagnostic delays. Dermoscopy, increasingly recognized as a non-invasive diagnostic tool for non-pigmented tumors, provides specific morphological clues [5, 6].

## Case report

A 56-year-old woman with a history of high-grade serous tubal carcinoma treated with hysterectomy and chemotherapy presented with cutaneous lesions evolving for one month. They initially appeared as a painful nodule and several papules on the abdominal surgical scar, then progressed to multiple firm, erythematous infiltrated nodules confluent into plaques on the lower abdominal wall and thigh roots.

**Figure 1:**
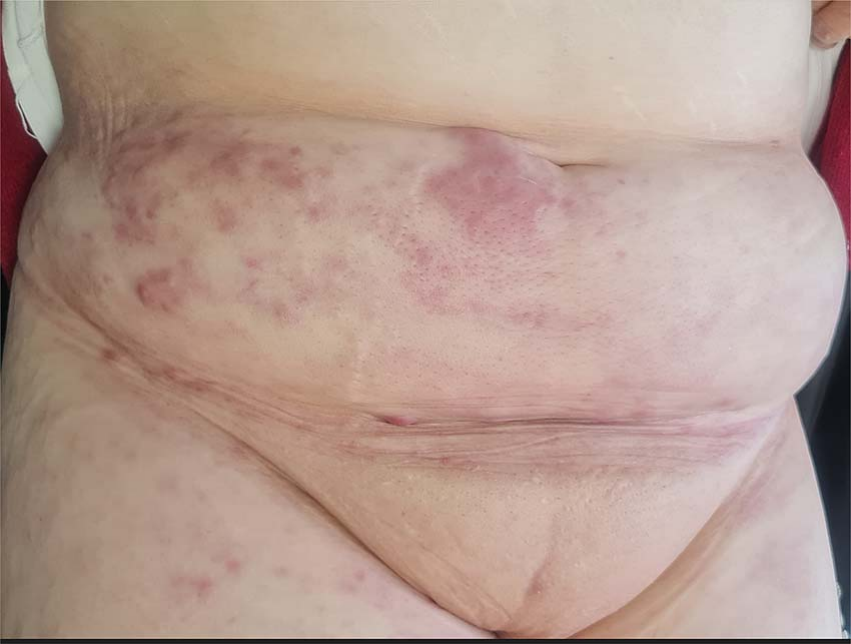
Multiple erythematous nodules and infiltrated plaques along the lower abdominal wall and surgical scar.

**Figure 2:**
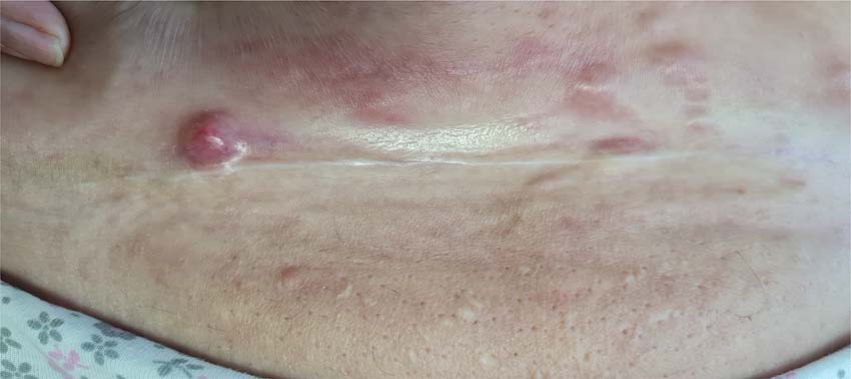
Close-up view showing a firm, erythematous, painful nodule developing directly on the hysterectomy scar.

**Figure 3:**
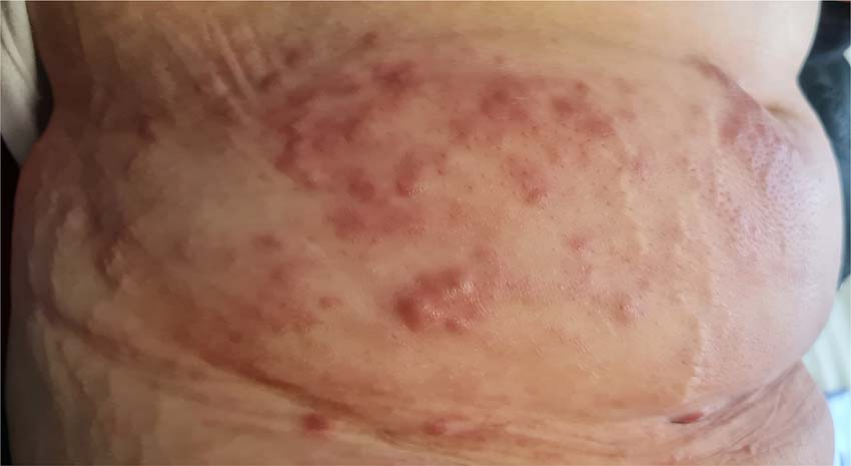
Erythematous infiltrated nodules confluent into plaques on the lower abdominal wall.

Examination showed painful erythematous nodules and plaques with peau d’orange changes and right lower-limb lymphedema (Fig. 1). A close-up revealed a firm, erythematous, painful nodule arising directly from the scar (Fig. 2), while additional infiltrated nodules extended across the abdomen (Fig. 3).

**Figure 4:**
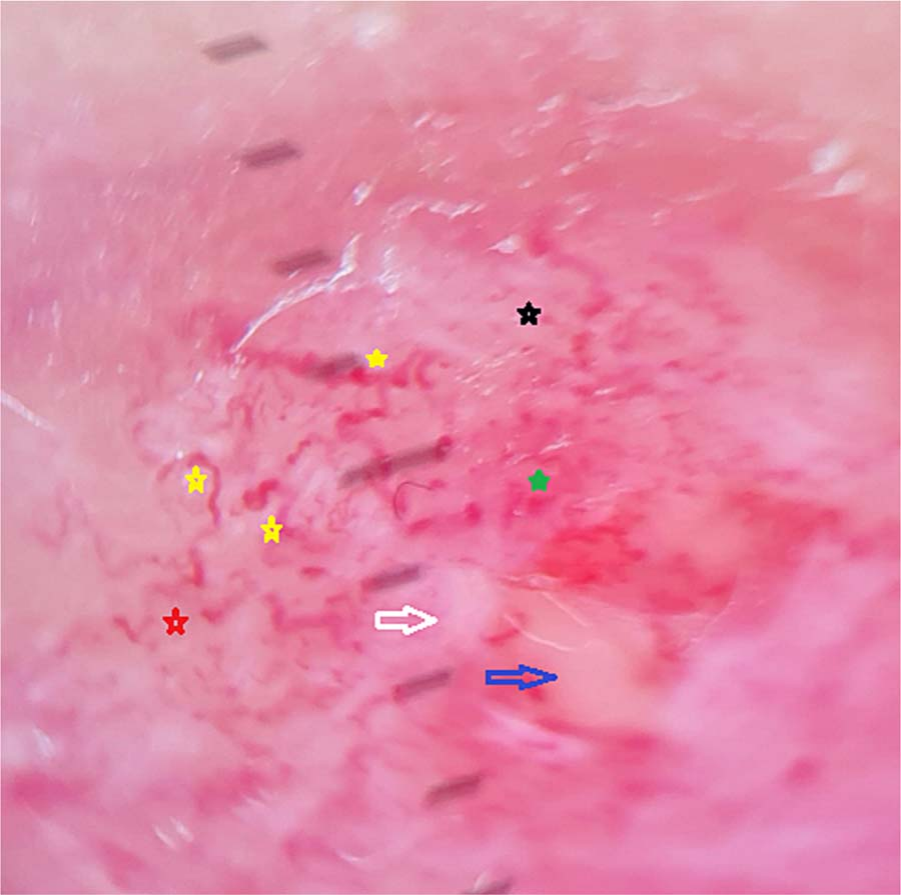
Dermoscopic image of the nodule on the hysterectomy scar showing a diffuse erythematous background. Yellow stars indicate serpentine vessels, red stars arborizing vessels, green stars glomerular vessels, black stars dotted vessels, white arrows white structureless areas, and the blue arrow a central yellow-orange zone.

Dermoscopy demonstrated a diffuse erythematous background with a polymorphic vascular pattern including irregular linear, arborizing, dotted, glomerular, and serpentine vessels, combined with whitish structureless areas and superficial hemorrhages (Fig. 4).

**Figure 5:**
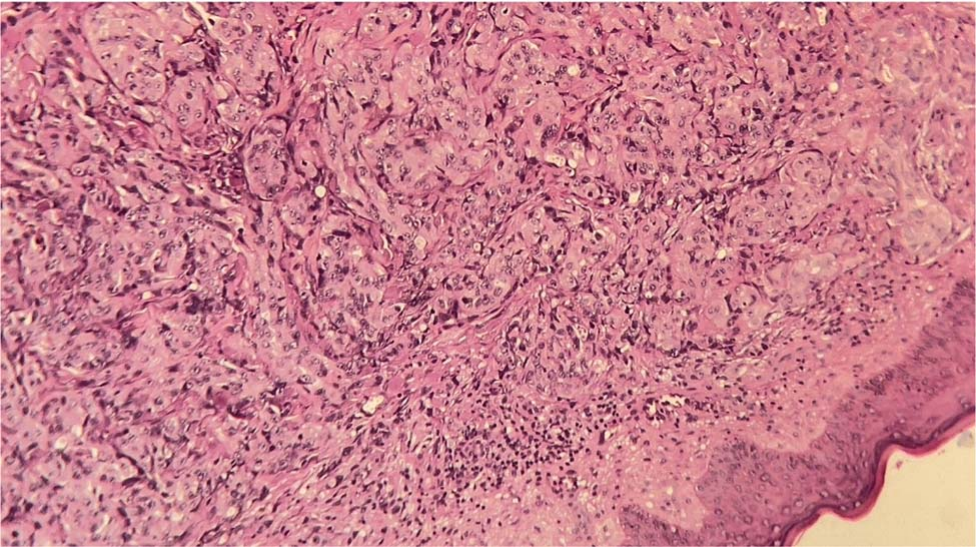
Histopathological section of the skin biopsy showing dermal infiltration by atypical epithelial cells arranged in nests and cribriform structures, with marked nuclear pleomorphism and preserved epidermis, consistent with metastatic high-grade serous carcinoma (H&E, ×200).

Skin biopsy revealed dermal infiltration by high-grade serous carcinoma, organized in nests and cribriform structures with pleomorphic cells, abundant cytoplasm, and hyperchromatic nuclei, within a fibrous stroma and with sparing of the epidermis (Fig. 5). Imaging confirmed peritoneal carcinomatosis with pelvic lymphadenopathy.

## Discussion

Our observation emphasizes surgical scars as potential sites of tumor recurrence. CM of ovarian or tubal origin are rare but documented [2, 3]. Most reported cases involve umbilical metastases (Sister Mary Joseph’s nodules) [7] or laparoscopic port-site metastases [4]. Development on a hysterectomy scar is unusual, making this case original and relevant.

Dermoscopy played a central role in diagnosis. Chernoff et al. [1] described hallmark dermoscopic features of CM, including diffuse erythema and polymorphic vascularization. Multicenter studies [5] confirmed the diagnostic value of irregular linear, arborizing, dotted, and glomerular vessels. In our case, serpentine vessels were also identified—dilated, sinuous structures reflecting anarchic neoangiogenesis. Rarely described in classical reports, their presence suggests they may represent an additional diagnostic sign. Their coexistence with other vascular morphologies reinforces the concept of chaotic angiogenesis as a universal feature of CM. Similar findings have been reported in metastases from esophageal adenocarcinoma [6], renal carcinoma [8], ovarian carcinoma [7], breast carcinoma [9], and nasopharyngeal carcinoma [10]. Dermoscopy thus helps differentiate CM from mimicking conditions such as cellulitis, panniculitis, hypertrophic scars, or dermatofibroma, and directs clinicians promptly to biopsy.

Histopathology remains the gold standard. In our case, biopsy confirmed dermal infiltration by high-grade serous carcinoma, showing cribriform structures, pleomorphic cells with abundant cytoplasm, nuclear atypia, and high mitotic activity, within fibrous stroma and sparing of the epidermis. This finding is important because it demonstrates secondary dermal involvement, unlike primary cutaneous tumors that often invade the epidermis [1, 3].

Prognostically, CM indicate advanced disease and poor outcomes. However, early detection is crucial, as it prevents misdiagnosis, alerts clinicians to recurrence, and allows timely therapeutic adjustment. Although systemic treatment remains the mainstay, early recognition of CM can improve quality of life through better symptom control and decision-making.

This case also has educational value, stressing the importance of careful clinical surveillance of surgical scars in patients with gynecologic cancers. We propose that dermoscopy should be systematically integrated into follow-up protocols to facilitate early detection of atypical cutaneous recurrences.

## Conclusion

Cutaneous metastases secondary to tubal carcinoma are exceptional, and their onset on a hysterectomy scar represents a rare but significant presentation. Dermoscopy, by demonstrating polymorphic vascular networks including serpentine vessels, provides a reliable non-invasive diagnostic tool. While histology remains indispensable, dermoscopy accelerates biopsy indication and prevents diagnostic delay. This case underscores the importance of systematic dermoscopic evaluation of surgical scars in gynecologic oncology follow-up.
